# Epidemiology of Viral Hepatitis and Liver Diseases in Mongolia

**DOI:** 10.5005/jp-journals-10018-1127

**Published:** 2015-01-06

**Authors:** Bira Tsatsralt Od

**Affiliations:** Department of Gastroenterology, National Institute of Medicine, Ministry of Health, Education and Science, Ulaanbaatar, Mongolia

**Keywords:** Hepatitis B virus, Hepatitis viruses, Mongolia, Genotype, Hepatitis E virus.

## Abstract

Mongolia which is located in Northern Asia between Russia and China is endowed with one of lowest population density in the world. Acute hepatitis due all types of hepatitis virus has been reported in Mongolia. Also, dual and triple hepatitis viruses, HBV, HDV and HCV are highly prevalent among patients with chronic liver disease living in Mongolia. Due to these facts, liver cancer is the leading cause of cancer mortality in Mongolia. The national immunization program including vaccination against hepatitis B was started in 1991 and screening of blood donations for HBsAg and anti-HCV was introduced in 1993 and 1997 respectively. The incidence of hepatitis viruses showing a downhill course in some parts of Mongolia, but comprehensive efforts are needed to control hepatitis viruses and containment of hepatitis related liver diseases and liver cancer in Mongolia.

**How to cite this article:**

Tsatsralt OB. Epidemiology of Viral Hepatitis and Liver Diseases in Mongolia. Euroasian J Hepato-Gastroenterol 2015;5(1):37-39.

## INTRODUCTION

Mongolia is located in Northern Asia and has one of the lowest population densities in the world. About two-thirds of the total population is under age 30 years; 28.5% of whom are under 14.^1^ Approximately, 50% of the population lives in cities, but many still live a nomadic lifestyle, and 36% of the population lives below the poverty line.^2^ Communicable diseases are one of the main health problems, with hepatitis comprising 41% of the reported infectious diseases in 1997.^3^ Economic activity in Mongolia has traditionally been based on herding and agriculture. Mongolia has extensive mineral deposits. Copper, coal, gold, molybdenum, fluorspar, uranium, tin, and tungsten account for a large part of industrial production and foreign direct investment. By 2008 estimates, agriculture makes up 18.8% of employment, industry 38.5% and services 42.7%. The 2008 estimate of unemployment was 2.8%. The purchasing power parity per capita is $3200.^2^ Mongolian citizens are provided free or low cost healthcare at government run hospitals. Private hospitals and clinics are also available for those who can afford them. Access to care is therefore universal, although diagnostic testing is often not performed for infectious diseases and cases are treated empirically. Gender-specific mortality rates were 750.8 per 100,000 males and 476.1 per 100,000 females. With regards to age, perinatal pathologies and diseases of the respiratory system were the main causes of mortality in 0 to 4 year-old, injuries and poisoning in 5 to 44-year-old, and diseases of the circulatory system and neoplasms in persons above 45 years.

According to the National Center for Communicable Diseases data analyses, cancer incidence and mortality has been increasing steadily for the last decade, and remain the second leading cause of population mortality.

**Table:** 

*Leoding couses 8f concer m8rtolity (in 2014)*									
*Couses*		*Percentage* *of cancer* *ortality*		*Per 10,000* *population*		*Per 10,000* *population*			
						*Mole*		*Femole*	
Liver cancer		44.0		4.7		5.6		3.9	
Gastric cancer		14.8		1.6		2.1		1.1	
Lung cancer		11.7		1.2		2.0		0.5	
Cervical cancer		7.2		0.6		–		0.6	

The age-specific incidence rates for 2004 demonstrated that viral hepatitis B was the most common in 10 to 44-year-old population (94.7%) and viral hepatitis A among aged 1 to 24 years (95.3%). The incidence of viral hepatitis A was 104.2 per 10,000 2-year-old, 129.9 per 10,000 3-year-old, 139.3 per 10,000 4-year-old and 58.6 per 10,000 5 to 9-year-old. In contrast, the incidence of viral hepatitis B increased by 1.1-1.8 per 10,000 in 15 to 24-year-old compared to the previous year, and reached 8.5 per 10,000 in 20 to 24 age group. Despite the comparatively low incidence of viral hepatitis B in age groups that received hepatitis B virus (HBV) vaccination, it was 11.1% in children under 14 ages.

Mongolia is divided into 18 provinces (aimags), then viral hepatitis has decreased in all aimags, 19.8 per 10,000 population in the last decade.

From all registered viral hepatitis, 82.2% (3959) cases were viral hepatitis A, 15.0% (720) cases were viral hepatitis B and 2.6% (127) cases were viral hepatitis C, the incidence of viral hepatitis B and C increases in 2003.

## STUDIES ABOUT VIRAL HEPATITIS IN MONGOLIA, ACCOMPLISHED AS COLLABORATIVE WORKS WITH JAPAN

Hepatitis viruses have been documented to account for a significant amount of acute hepatitis with a recent study showing 16.4, 32.7, 1.8, 27.3 and 6.4% of acute hepatitis caused by HAV, HBV, and hepatitis delta virus (HDV) coinfection, HDV super-infection and hepatitis C virus (HCV) respectively.^4^ According to NCCD statistics for 2013, reported patients with acute hepatitis A (n = 1635), B (n = 524), C (n = 80) and D (68) have been noted. These may be compared with the figures of about 3 months of 2010: A (n = 338), B (n = 138), C (n = 28) and D (16). They do not have the capability to diagnose hepatitis E virus (HEV).

Two hundred and eighty-nine first time and 114 repeat donors at the Blood Center of Mongolia (MBC) were tested for serological and molecular markers of HBV, HCV and HDV infections. Among the 403 blood donors, 33 (8.2%), 21 (5.2%), and 27 (6.7%) tested positive for hepatitis B surface antigen (HBsAg) and/or HBV DNA, HCV RNA, and HDV RNA respectively.^1^

We also tested for serological and molecular markers of HBV, HCV and HDV infections in patients with viral hepatitis. Of the 207 patients, 144 (69.6%), 106 (51.2%), and 117 (56.5%) tested positive for hepatitis B surface antigen (HBsAg) and/or HBV DNA, HCV RNA, and HDV RNA, respectively. Collectively, 172 patients (83.1%) were viremic for one or more of these viruses, including dual viremia of HBV/HDV (26.6%) or HBV/HCV (7.7%) and triple HBV/HCV/HDV viremia (30.0%). Of note, triple ongoing infection was significantly more frequent among patients with hepatocellular carcinoma than among those with chronic hepatitis (63.2 *vs* 14.4%, p < 0.0001).^2^

There are three published studies that have tested for the presence of HEV in humans in Mongolia. The first one tested 249 healthy people from 23 to 86 years of age including city dwellers and nomads, and found that 11% had antibodies to HEV.^3^ There was no appreciable difference between city dwellers and nomads, or between age groups, with the exception of the 23 to 29 years group which only had 4% positive. A second study prospectively tested patients from 16 to 48 years of age with acute hepatitis during two winter months (December and January) in Ulaanbaatar for IgM and IgA against HEV.^5^ Of 110 patients, none were positive. The third study tested 717 healthy children from birth to 20 years of age for IgG antibody to HEV and found antibody in only 5 (0.7%).^6^

The differences between these studies are not surprising. In Nepal, where HEV is endemic and causes large outbreaks, the seropositivity rate is very low in children, and does not start to appreciably rise until the teen years, with the greatest increase between the ages of 20 and 40. The rate found in adults in the first study is probably more characteristic of this population, and also demonstrated a lower rate in the 23 to 29 years age group.^6^ The study on acute hepatitis is limited by the fact that testing was only performed when it would be less likely for this enterically transmitted pathogen to be circulating in the winter months. In addition, they only tested for IgM and IgA, which can be more difficult to detect and they did not test acute and convalescent sera.

Surprisingly, a recent study of pigs from four swine farms near Ulaanbaatar revealed 91.8% to be positive for HEV antibodies and 36.6% had detectable HEV RNA.^7^ They were classified in two novel phylogenetic groups within genotype 3. No reports to date have determined the risk of HEV in swine farmers, but it is likely that the pigs could be a source of infection to humans.
